# Feline communication strategies when presented with an unsolvable task: the attentional state of the person matters

**DOI:** 10.1007/s10071-021-01503-6

**Published:** 2021-04-02

**Authors:** Lingna Zhang, Katie B. Needham, Serena Juma, Xuemei Si, François Martin

**Affiliations:** Nestlé Purina Research, Saint-Louis, MO USA

**Keywords:** Feline, Cat, Social referencing, Showing, Cognition

## Abstract

Research on social cognitive ability in domestic cats is limited. The current study investigated social referencing in cats when exposed to first, a solvable, and then, an unsolvable scenario (i.e., reachable and unreachable treats) in the presence of either an attentive or an inattentive caregiver. Cats expressed more gaze alternation (*P* = 0.013), but less interaction with the caregiver (*P* = 0.048) and approached the treat container less frequently (*P* = 0.017) during the unsolvable test, compared to the solvable test. When in the presence of an attentive caregiver, cats initiated first gaze at the caregiver faster (*P* = 0.001); gazed at the caregiver for longer (*P* = 0.034); and approached the treat more frequently (*P* = 0.040), compared to when the caregiver was inattentive. Significant interaction was observed between test and caregiver’s attentional state on the expression of sequential behavior, a type of showing behavior. Cats exhibited this behavior marginally more with attentive caregivers, compared to inattentive caregivers, but only during the unsolvable test. There was a decrease in sequential behavior during the unsolvable test, compared to solvable test, but this was only seen with inattentive caregivers (*P* = 0.018). Our results suggest that gaze alternation is a behavior reliably indicating social referencing in cats and that cats’ social communication with humans is affected by the person’s availability for visual interaction.

**Supplementary Information:**

The online version contains supplementary material available at 10.1007/s10071-021-01503-6.

## Introduction

Domestic cats (*Felis silvestris catus*) have traditionally been considered solitary (Driscoll et al. 2007; Velli et al. 2015). However, the social cognition capability of domestic cats may have been underestimated. Cats show social flexibility, as demonstrated by their ability to live solitarily or to form social groups, depending on factors such as distribution of resources and social experiences (Vitale Shreve and Udell 2015). Domestic cats are a popular companion animal (AVMA 2017–2018; Euromonitor International 2019), suggesting that they are successful in sharing a living space and communicating with humans. Using a method established in human infants, a recent study demonstrated that cats show a capacity similar to those of dogs and infants to form distinct attachment styles towards human caregivers (Vitale et al. 2019). Research focused on the socio-cognitive capacity of domestic cats in the context of human-cat interaction has recently increased (Vitale Shreve and Udell 2015). Some evidence of successful vocal communication between cats and humans include, (1) the meowing of domestic cats is more pleasant than that of African wild cats to a human listener (Nicastro 2004), (2) cats emit specific solicitation purring to the owner at feeding (McComb et al. 2009), (3) cats are able to differentiate between an owner and a stranger’s voice (Saito and Shinozuka 2013), and distinguish their name from similar sounding words (Saito et al. 2019), as well as to match a human face to the corresponding voice (Takagi et al. 2019). A succession of half-blinks followed by a prolonged eye narrowing or closure, also known as slow blink sequence, has been suggested to facilitate positive emotional communication between cats and humans (Humphrey et al. 2020). Pet cats are also able to recognize human attentional state (Mertens and Turner 1988; Ito et al. 2016; Vitale and Udell 2019), read human emotional expression (Merola et al. 2015; Galvan and Vonk 2016; Quaranta et al. 2020), and use human-directed cues, such as pointing (Miklósi et al. 2005; Kraus et al. 2014) and gazing (Pongrácz et al. 2019), to locate hidden food, Cats can also use social referencing when confronted with an ambiguous item (e.g., running fan with plastic ribbons). As defined by Merola et al. (2015), social referencing is a process where “animals look at humans (informer) when facing unfamiliar situations that are difficult to interpret, and act in accordance with the informer’s positive or negative emotional reactions”.

Little is known about if and how cats intentionally provide information to humans (i.e., showing behavior). Showing is a form of functionally referential communication that consists of animals (informer) referring to a desired external event/target (directional component) and attracting the attention of humans (attention-getting component) to the event/target (Miklósi et al. 2000). Gaze alternation (i.e., successively looking at the receiver and the desired target when faced with an unsolvable situation) is a common showing behavior in a variety of species, such as human infants (Bruinsma et al. 2004), chimpanzees (Leavens and Hopkins 1998), and squirrel monkeys (Anderson et al. 2007). Pointing is also used by humans and chimpanzees while showing (Blake et al. 1994; Leavens et al. 1996; Leavens and Hopkins 1999). In species without hands, behaviors that serve a similar function to pointing have been observed. Showing behavior in domestic dogs, a species with a similar human-animal connection to that of the domestic cat, has been well studied (Miklósi et al. 2000; Gaunet and Deputte 2011; Heberlein et al. 2016; Savalli et al. 2016). In addition to gaze alternation, dogs use spatial positioning of their body to indicate target objects located at different heights (Gaunet and Deputte 2011; Marshall-Pescini et al. 2013). Sequential behaviors (e.g., approach human/target$\to$  look at human $\to$ approach human/target$\to$ look at target) were observed in a comparative study evaluating showing behavior of dogs and wolves subjected to out-of-reach food, in the presence of a cooperative or competitive human partner (Heberlein et al. 2016). Worsley and O’Hara (2018) identified 19 referential gestures expressed by domestic dogs during daily owner-dog interaction under various communicative contexts, such as “give me food/drink” or “get my toy/bone.” The expression of showing behavior in dogs can be affected by the owner’s attentional posture (i.e., their availability to make eye contact; Savalli et al. 2016), by their former experience with agility and obedience training (Udell and Brubaker 2016), and their rearing environment (i.e., kennel versus household; Udell and Brubaker 2016). Human attentional states were also shown to influence the production of communication signals in non-human primates (Hostetter et al. 2001; Hattori et al. 2009; Leavens et al. 2009; Bourjade et al. 2014; Canteloup et al. 2015).

Miklósi et al. (2005) compared the referential communication of cats to those of dogs with humans, when presented with an unsolvable task. Their results suggest that cat communication lacks some attention-getting components of showing due to a delayed and reduced number of instances of gaze behavior towards humans “for help,” as well as fewer instances of gaze alternation, when compared to dog communication (Miklósi et al. 2005). The less recognized showing behaviors used in other dog studies (e.g., spatial positioning, sequential behaviors; Heberlein et al. 2016; Worsley and O’Hara 2018) were not included and the potential differences between the two species’ communication patterns with humans were not considered. To date, research focusing on the effect of a human’s attentional state on expression of referential communication by cats is lacking. In the current study, we sought to investigate (1) the expression of showing behavior in cats, by comparing their response when presented with a solvable task, followed by an unsolvable task, and (2) if a human’s attentional state affected showing behavior in cats. We also aimed to identify components of showing behavior that are typical of cats by creating an ethogram.

## Methods

### Subjects

A total of 75 healthy, purpose-bred, mixed-breed, neutered cats (30 females; 45 males), aged between 1 and 12 (7.83 ± 2.83) years, with body scores between 4 and 7 on a 9-point scale (Laflamme 1997), were included in this study. The cats were individually or pair-housed (1.4 × 1.4 × 2.5 m) with visual access to the neighboring cats and the outdoors. Additionally, all cats had access to a shared activity room (12.1 × 2.1 × 2.5 m) for 2–3 h per day with a stable social group of up to 8 cats. Each cat received regular sessions of individual socialization time with their designated caregivers (e.g., playing, grooming, cuddling). Cats were fed daily before 10:00 h and their participation in the study occurred in the afternoon between 12:30 h and 15:00 h.

### Cat behavior laboratory

**Fig. 1:**
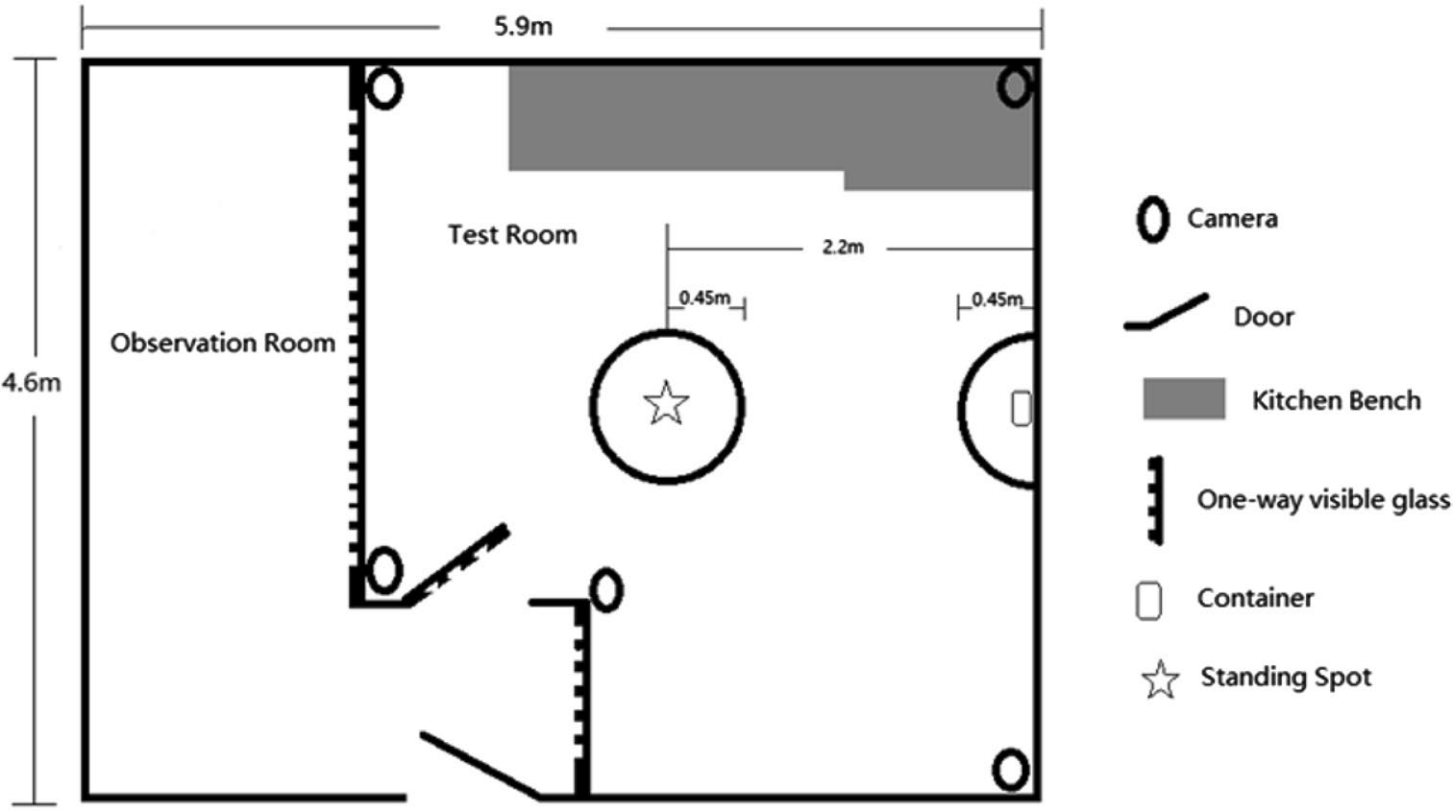
Cat behavior laboratory composed of an observation room and a test room

Acclimation, training, and testing all took place in the same cat behavior laboratory (set-up is shown in Fig. 1). A plastic container (17 cm × 12 cm × 6 cm) with lid was placed at one side of the room along the wall, secured to the floor, and a standing spot was designated on the floor for the caregiver 2.2 m away from the container. Areas around the container and standing spot (≤ 0.45 m) were marked with tape to assist with behavioral coding. The one-way glass mirror on both the wall and the door of the test room were covered with white contact paper to block any reflections that might distract the cats. During acclimation, training, and testing, soft classical music (average of 45 decibels, or the equivalent of a quiet office) was played in the observation room, serving as background noise. Soft music is regularly played in the cattery as auditory enrichment and the cats are used to it. Study has reported no difference between classical music and silence (i.e., no music) on physiology and behavior of cats at veterinary clinic (Hampton et al. 2020). Cats were video-recorded with Mangold’s VideoSyncPro software (Mangold International GmbH, Arnstorf, Germany) using four wireless video cameras (Bosch 540 TVL hi-performance day/night cameras, Bosch GmbH, Gerlingen, Germany, equipped with RF-Link 5.8 GHZ SR wireless transmitter/receivers, RF-Link/Araneus USA, Inc., Corona, California, USA) during both the solvable and unsolvable tasks.

### Procedure

The cats’ behaviors were observed in two situations (attentive and inattentive caregivers) during two experimental phases (solvable and unsolvable tasks).

#### Pre-selection

An initial group of 75 cats (30 females; 45 males) were selected for the study. All cats were subjected to the following sequence and their behavior observed: (1) approach while in primary enclosure, (2) remove from primary enclosure, (3) put in a carrier for a short period (less than a minute), (4) remove from carrier and hold securely, (5) place on a table, (6) pet, (7) brush, (8) simulated physical examination (palpate abdomen, lift tail, touch ears, and open mouth), and (9) nail trim. Cats were given a treat at the end of the interaction. Cats that showed stress-related behavior (e.g., crouched posture, pupil dilation), that did not positively engage with the technician (e.g., moving away from the person), or did not consume the treat were removed from the study (*n* = 19).

#### Acclimation

**Fig. 2:**
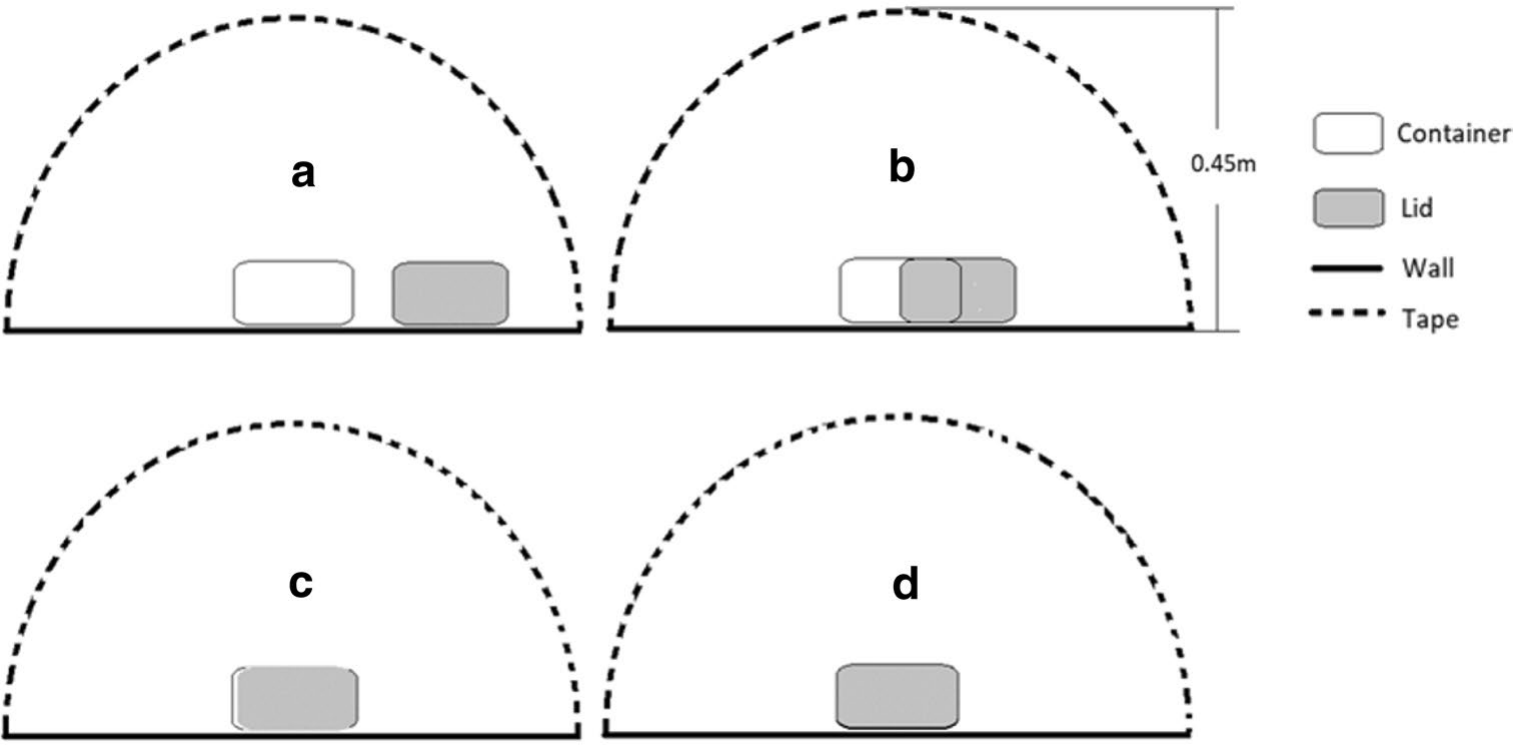
Container is covered in four different ways: **a** uncovered with lid to the side, **b** covered half-way, **c** mostly covered but not sealed, and **d** fully covered with lid sealed

Out of the original 75, a total of 56 cats (21 females; 35 males) were selected for the acclimation phase. Two female caregivers, familiar to the cats, acclimated, trained, and tested the cats. Cats were randomly divided into two groups and each assigned to a caregiver. Once a day, for 3 days, cats were individually brought to the cat behavior laboratory for a play session that lasted approximately 10 min. During the play session, the caregiver interacted with and treated the cat from an uncovered container (Fig. 2a). Each cat was given five opportunities to be treated from the container per session, as training for the test phase. Two different commercial dry and soft cat treats were used depending on the cats’ preference. A cat would enter the training phase only if a treat was consumed from the container four out of five times on day 3 of acclimation (7 females; 24 males). A container unique to each individual was utilized for the acclimation, training, and test. The testing room was spot-cleaned between cats to minimize the presence of scents from unfamiliar individuals.

#### Training

On the training day, the following procedure was followed for each individual:

- The experimenter set up the container as in Fig. 2a at the designated location and exited the testing room.
- The caregiver brought an individual cat into the testing room using a pet carrier, opened the carrier, and directed the cat towards the container with calls and gestures.
- The caregiver showed a treat to the cat, placed the treat inside the container, while leaving it uncovered. The cat was not allowed to eat the treat at this point.
- The caregiver then picked up and petted the cat, walked to the designated standing spot, and placed the cat on the ground, facing the direction of the container. The caregiver remained standing in the designated spot and visually followed the cat, without physical or verbal interaction. The trial concluded 2 min after the cat was released by the caregiver after she placed the treat in the container. Cats that did not successfully retrieve the treat from the container within 2 min were directed towards the container and encouraged to eat the treat (i.e., caregiver would stand near the container, call the cat, and point to the treat).
- Steps “c” to “d” were repeated an additional four times, each time with modified lid positions in the order of b-b-c–c in Fig. 2. At no point during training was the container fully sealed. The caregiver sham-covered the container each time by gently pressing on the lid. Between trials, the caregiver provided 30 s of positive interaction to the cat, without treating (e.g., talked to, visually followed, or petted the cat upon approach).

The cats were selected to enter the test stage only if they successfully retrieved a treat from the container during the last two consecutive training sessions (6 females; 23 males).

#### Test

On the next day, cats experienced the solvable task, followed by the unsolvable task. For both tasks, key steps were similar to training, except that the container was covered with the lid not sealed (solvable task) or sealed (unsolvable task) as shown in Fig. 2c, d. The solvable task included steps “a” to “d” of training, and the unsolvable task included steps “c”–“d,” with a 1-min break between each task, during which the caregiver interacted positively with the cat (e.g., talked to, visually followed, or petted the cat upon approach). During the 2-min period of standing at the designated spot in both tasks, caregivers were instructed to be available for visual interaction with the cat by looking in its direction (attentive state), or not be available for visual contact by staring at the stopwatch that was held close to their chest (inattentive state). Each cat experienced the solvable task first, and then the unsolvable task. The attentional state of the caregiver was the same during both tasks for the same cat, and the cat was only exposed to one of the two attentional states (i.e., attentive or inattentive). Which cat received which attentional state was determined with pseudo-randomized ordering and, as a result, each caregiver displayed attentive state towards half of her assigned cats and displayed inattentive state towards the other half.

To pass the test phase, a cat had to eat the treat from the container during the solvable task and interact with the container (defined in Table 1) during the unsolvable task. Twenty-six cats (5 females; 21 males) passed the test. Three of the five female cats (aged 11.5, 5.58, and 5.19 years) that passed the test were assigned to the attentive state group and the two other female cats (aged 6.28 and 11.6 years) were assigned to the inattentive state group. Out of the 21 male cats that passed the test, ten cats (aged 8.30 ± 2.77 years) were assigned to the attentive state group and the other 11 cats (aged 8.19 ± 3.46 years) were assigned to the inattentive state group.

**Table 1:** Behavioral variables coded during the solvable and unsolvable tasks (Miklósi et al. 2000, 2005; Gaunet 2008; Marshall-Pescini et al. 2013; Heberlein et al. 2016)

Behavioral Variable	Definition
Interaction with container	*Duration* amount of time any frontal body part of the cat touches container (e.g., actively sniffing or pawing at container), excluding treat consumption
In proximity to container	*Latency* time elapsed from when cat is released until a front paw first enters taped area near container (≤ 45 cm; Fig. 1) *Duration* amount of time cat spends in taped area near container. Starts when a front paw enters taped area and ends when last hind paw exits taped area *Frequency* number of times cat is in proximity to container
Gaze at container	*Duration* amount of time cat looks toward container with head still, without approaching container *Frequency* number of times cat gazes at container
Gaze at caregiver	*Latency* time elapsed from when cat is released until first gaze at caregiver *Duration* amount of time cat turns/lifts head towards caregiver’s face/head until cat turns head away *Frequency* number of times cat gazes at caregiver
In proximity to caregiver	*Duration* amount of time cat spends near (≤ 45 cm area) caregiver’s designated standing spot (Fig. 1). Starts when a front paw enters taped area and ends when last hind paw exits taped area *Frequency* number of times cat walks to caregiver
Contact with caregiver	*Duration* cat approaches and establishes physical contact with any body part (excluding the tail) of caregiver (e.g., rubbing, pawing, climbing on legs)
Gaze alternation	*Frequency* cat directs gaze continuously (within 2 s) from caregiver (look at the face/head) to container (or vice versa)
Vocalization	*Frequency* number of times cat meows
Sequential behavior	*Frequency* Cat continuously gazes or is in close proximity to the container or caregiver. Excludes gaze alternation (1) Caregiver to container: cat gaze at caregiver and/or is in proximity to caregiver followed by cat gaze at container and/or is in proximity to container (2) Container to caregiver: cat gaze at container and/or is in proximity to container followed by cat gaze at caregiver and/or is in proximity to caregiver

Cats that failed acclimation, training, or the test (1) showed stress-related behavior in the novel environment, (2) did not show interest in the treat, or (3) did not interact with the lid.

### Data collection and analysis

#### Behavior coding

Cats’ behavior during the 2 min post-release for both the solvable and unsolvable tasks was cataloged and coded with Mangold’s INTERACT 9 software (Mangold International GmbH, Arnstorf, Germany). An ethogram of the coded behaviors is presented in Table 1. Five of the 26 videos from cats that passed the test were chosen randomly and coded for inter-rater reliability. The average Cohen’s Kappa for all coded behaviors was greater than 0.92.

### Statistical analysis

The effects of factors (i.e., caregiver, cat’s weight, sex, and age) on cat performance (i.e., pass or fail) during the acclimation-training-test process were analyzed with Chi-square analysis, point-biserial correlation (which is equivalent to Pearson correlation), and simple t test. Effect size was calculated using Cohen’s *d*.

Data were analyzed to compare the behavior of cats between the solvable and unsolvable task (test type) and between attentional states of caregivers (attentional type) using generalized Linear Mixed models in SAS 9.3 (SAS Institute Inc., Cary, NC, USA). Least square means and standard errors were reported for each measure. Caregiver effect was not included in the models due to non-significant effect. Sex was removed from the final model due to the skew towards male cats in the study. Cat age was grouped into two categories, younger (1–7 years) and older (8–12 years; Vogt et al. 2010). The original models included test type, caregiver’s attentional type, age of cat, and interactions between and among age, test, and attentional type, as dependent variables, and randomized by cat. For variables not affected by interactions between or among effects, non-significant interactions were gradually removed from the final models. Pairwise difference was considered as significant with *P* < 0.05, and a trend at 0.05 ≤ *P* < 0.10 from t test after simulated adjustment.

## Results

### Effect of sex on cat performance

Males were more likely (*χ*^2^ = 6.91, *df* = 1, *P* = 0.009) to pass the acclimation-training-test process than females (Table 2). Body weight (*ρ* = 0.29, *P* = 0.032), but not age (*ρ* = 0.10, *P* = 0.449) of cat, was positively correlated with the chance of a cat passing or failing the test. The effect of body weight (*t*_54_ = 2.20, *P* = 0.032) on cat performance was confounded by sex, because male cats are generally heavier than females (t_54_ = 6.99, *P* < 0.001).

**Table 2:** Performance of cats, by sex

Cat sex (#)	Pass # (rate)	Fail # (rate)
Female (*n* = 21)	5 (23.8%)	16 (76.2%)
Male (*n* = 35)	21 (60.0%)	14 (40.0%)

Pass: cat completed the entire acclimation-training-test protocol

### Effect of age on cat behavior

Older cats spent marginally less time in contact with (8.46 ± 5.24 s vs 26.2 ± 6.65 s; *t* = − 2.08, *P* = 0.051), and in proximity to the caregiver (23.4 ± 6.18 s vs 43.0 ± 7.83 s; *t* = − 1.95, *P* = 0.062). They approached the treat container more frequently (2.74 ± 0.24 time vs 1.77 ± 0.30 time; *t* = 2.49, *P* = 0.021), compared to younger cats. The effect size of age on the three measures was relatively strong as indicated by Cohen’s *d* of 0.75, 0.69, and 0.63 respectively. Cat’s age or age-involved interactions did not significantly affect other measures.

### Cat behavior during the test

**Fig. 3:**
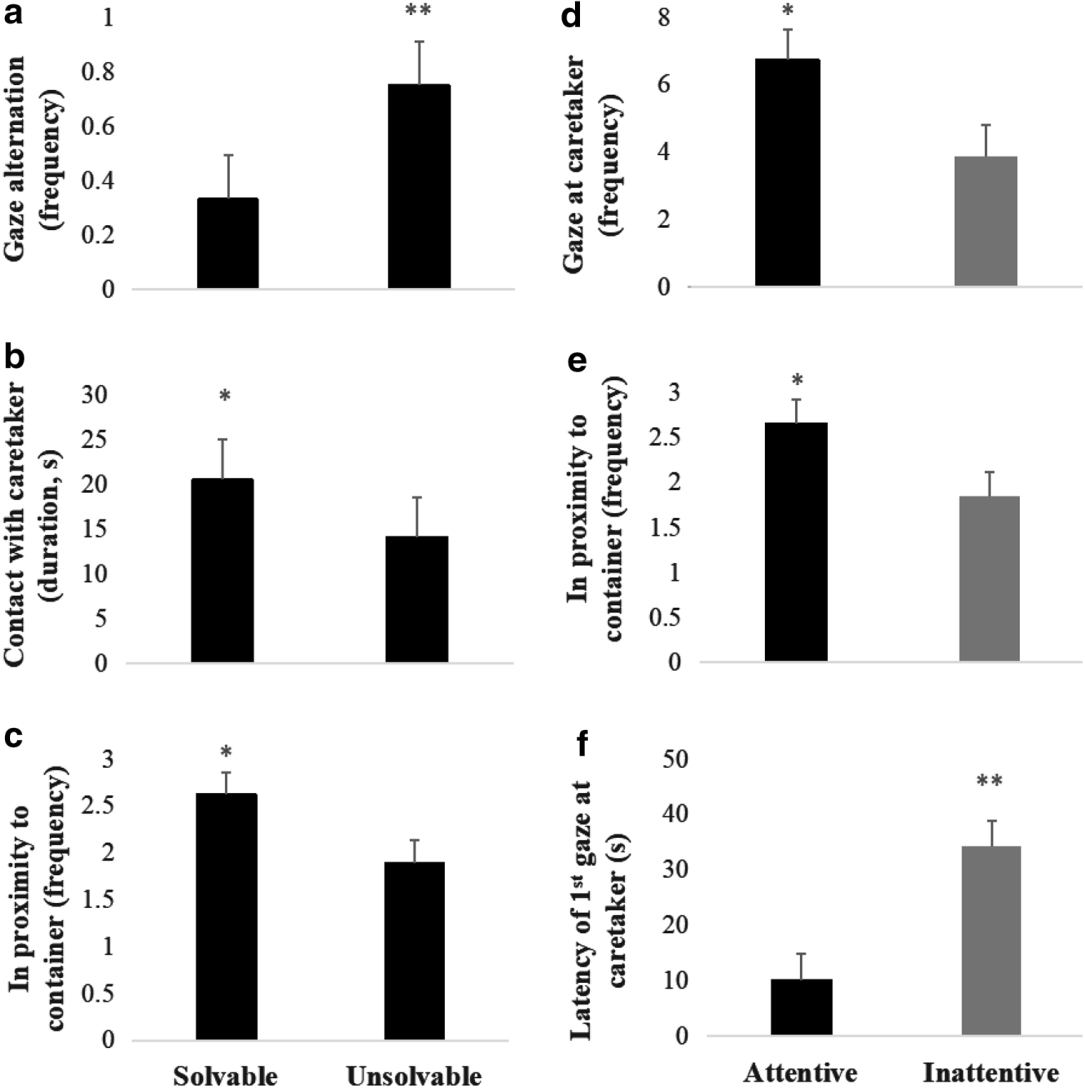
Significant effect of test type (solvable vs unsolvable) and caregiver’s attentional type (attentive vs inattentive) on behavioral measures in cats (*n* = 26). *, ** Least square means differed between treatment groups at 0.01 ≤ *P* < 0.05 and *P* < 0.01 based on simulated adjustment of the *t* test. The error bars indicate standard errors of least square means

Cats exhibited more gaze alternation (0.75 ± 0.16 time vs 0.33 ± 0.16 time; *t* = 2.67, P = 0.013; Fig. 3a), spent less time in contact with the caregiver (14.1 ± 4.47 s vs 20.5 ± 4.47 s; *t* = − 2.09, *P* = 0.048; Fig. 3b), and were in proximity to container less frequently (1.89 ± 0.24 time vs 2.62 ± 0.24 time; *t* = − 2.56, *P* = 0.017; Fig. 3c), during the unsolvable test, compared to the solvable test. The effect size of test type was small for gaze alternation (*d* = 0.14) and time spent in contact with the caregiver (*d* = 0.28), and moderate for frequency cats spent in proximity to the food container (*d* = 0.55). Test type did not significantly affect other measures.

Cats gazed at the caregiver more often (6.74 ± 0.88 s vs 3.88 ± 0.92 s; *t* = 2.26, *P* = 0.034; Fig. 3d), approached the container more frequently (2.66 ± 0.26 time vs 1.85 ± 0.27 time; *t* = 2.12, *P* = 0.040; Fig. 3e), and the latency of the first gaze at the caregiver was shorter (10.1 ± 4.72 s vs 34.3 ± 4.62 s; *t* = − 3.68, *P* = 0.001; Fig. 3f) when the caregiver was attentive compared to when the caregiver was inattentive. The effect size of the attentional state was moderate for frequency of gazing at the caregiver (*d* = 0.50) and frequency of approaching the container (*d* = 0.49), and very strong for latency of the first gaze at the caregiver (*d* = 0.96). The caregiver’s attentional state did not significantly affect other measures.

**Fig. 4:**
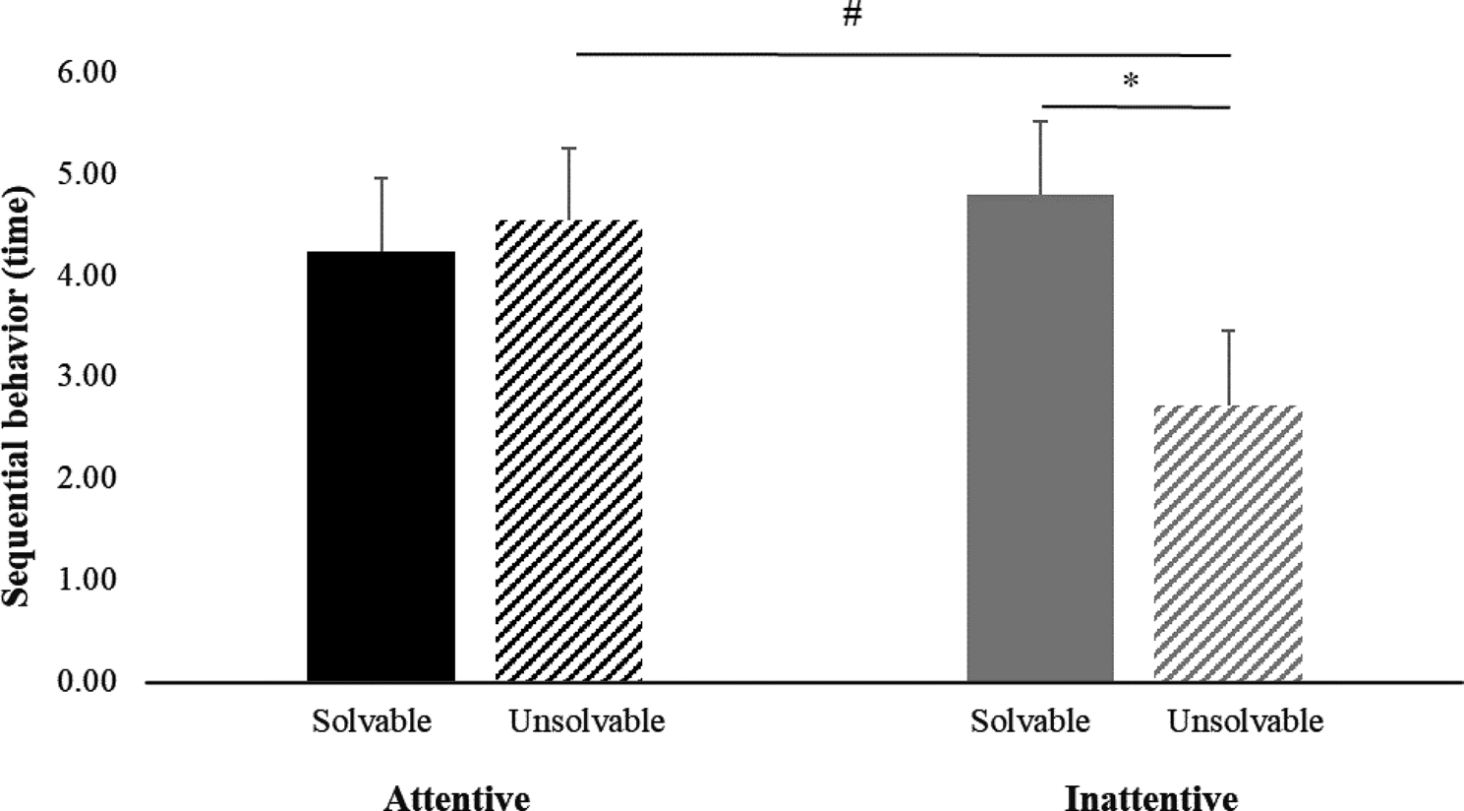
Significant effect of the interaction of test type and caregiver’s attentional type (ANOVA, *P* = 0.047) on the expression of sequential behavior in cats (*n* = 26). ^#^, * Least square means differed between treatment groups at 0.05 ≤ *P* < 0.10 and *P* < 0.05 based on simulated adjustment of the *t* test. The error bars indicate standard errors of least square means

A significant interaction between test and caregiver’s attentional types was only observed on the expression of sequential behavior (Fig. 4), not on other measures. Within the unsolvable test, cats exhibited marginally more sequential behavior in the presence of an attentive caregiver compared to inattentive caregiver (4.54 ± 0.71 time vs 2.72 ± 0.73 time; *t* = 1.80, *P* = 0.088), and the effect size of the attentional state was relatively strong (*d* = 0,71). When the caregiver was inattentive, cats expressed fewer instances of sequential behavior during the unsolvable test compared to the solvable test (2.72 ± 0.73 times vs 4.79 ± 0.73 times; *t* = − 2.57, *P* = 0.018), with a strong effect size (*d* = 0.80).

## Discussion

Despite the popularity of domestic cats as companion animals (AVMA 2017–2018; Euromonitor International 2019), research focused on cat social behavior, especially on human-cat interaction and factors influencing the relationship, remains relatively scarce (Vitale Shreve and Udell 2015). A better understanding of the socio-cognitive capacity of cats in this context may promote positive human-cat interactions and the forming of bond, and, ultimately, increase cat’s welfare. Our study is the first to investigate how caregiver’s attentional state affects human-directed communication behavior in cats when facing a solvable task (i.e., easily accessible treat) and an unsolvable task (i.e., inaccessible treat). Our results show that cats behave differently in these two situations. We also found that the attentional state of the human influenced the cat’s behavior during these tasks. The socio-cognitive capacity of domestic cats may be greater than previously assumed.

In our research, cats showed significantly more gaze alternation during the unsolvable test compared to during the solvable test, similar to what is reported in human infants (Bruinsma et al. 2004), and dogs (Miklósi et al. 2000; Savalli et al. 2014). Miklósi et al. (2005) studied cat showing behavior in a similar setting, but they focused on comparing behaviors between species (cat and dog) only during the unsolvable task rather than between the two test types within the same species. This prevented us from directly comparing the results and findings between the Miklósi et al. (2005) study and our study. However, it is worth mentioning that the cats in our study exhibited gaze alternation at a comparable level (0.48 ± 0.13 times) within the first minute during the unsolvable test (approximately 0.5 times in Miklósi et al. 2005). Albeit not statistically significant, cats also showed more sequential behavior during the unsolvable test when the caregiver was attentive. In addition to gaze alternation, dogs that face challenges (e.g., food/toy out of reach) increase their expression of other showing or attention-seeking behaviors, such as change of spatial location (Gaunet and Deputte 2011), sequential behaviors (Heberlein et al. 2016), and referential gestures (Worsley and O’Hara 2018). Based on these studies, we hypothesized that cats would express more social/attention-seeking behaviors during the unsolvable situation. Surprisingly, cats were in contact with the caregiver for longer periods and approached the container more frequently, and displayed sequential behavior more often with an inattentive caregiver during the solvable test compared to the unsolvable test. This may have been because, in our study, the caregiver was also the person giving the treats. The confounding effects of the association between human and desired objects (i.e., toys or treats) on pet communication behaviors have been reported previously. In a similar test where the owner acted as both toy hider and signal recipient, dogs showed no difference in certain attention-seeking behaviors (i.e., vocalizations, contacts, noisy mouth lickings, and gaze at the owner) when the owner was present without the toy and when both owner and toy were present in the room (Gaunet and Deputte 2011). Another study reported that when the owner took on the role of the experimenter, the dogs were more successful at informing their owners of the location of an object they wanted (Kaminski et al. 2011). Future studies should consider the inclusion of both “caregiver” and “experimenter” that are familiar to the cat, but maintain separate roles to reduce the association of caregiver and treating. Attention-seeking behaviors, such as sequential behavior and being in contact with caregiver were present during both test types in our study, but may have been driven by different motivations. During the solvable test, cats may have been motivated to exhibit showing/attention-seeking behavior after they successfully retrieved the treat to get more. However, during the unsolvable test, cats may have exhibited showing behavior because they were not successful at accessing the treat and they were trying to get the caregiver’s attention.

Cats in our research approached the treat container more frequently, took their first gaze at the caregiver sooner, and gazed at her more often when she was attentive. Previous studies reported that cats are more likely to beg for food when a human looks at or calls to them (Ito et al. 2016), and spend significantly more time in proximity to the attentive human, whether in a home or shelter environment (Vitale and Udell 2019). In addition, in our research, cats also decreased the expression of sequential behavior when the caregiver was inattentive compared to when she was not, and only during the unsolvable test. Our results align with these studies and provide additional evidence supporting that cats can recognize an owner’s attentional state, and when presented with an unsolvable task, can and will adjust their attention-seeking behaviors accordingly. Dogs also increase visual communicative behaviors (e.g., gaze alternation and sustained gaze) when they establish eye contact with owners compared to when the owners are not visually attending (Savalli et al. 2016). Explaining this communication strategy in dogs, and contrasting it with the fact that captive primates don’t resort to it, Savalli et al. (2016) proposed that it may reflect the phylogenetic and ontogenetic history of pet dogs (Miklósi and Topál 2013) and called for additional research on that matter. The results of our study on domestic cats lend support to the effect of domestication and socialization on this communication strategy.

Although interesting, our results regarding the behavioral differences observed in the cats when the person was attentive vs. inattentive must be interpreted with caution. Each cat was only exposed to one of the two attentional states (i.e., attentive or inattentive) and similar experimental design was used in dogs by Marshall-Pescini et al. 2013. This is a limitation of our study because even if they were randomly assigned, there could have been some characteristic of cats in the attentive group that made their results different from those in the inattentive group. It was not practical for the same cat to receive both attentional states. By doing so, cats would have experienced the unsolvable task twice. It was important to expose the cats to the unsolvable task only once in our experimental procedure to eliminate the confounding effect of learning since the main goal of the study was to compare cats’ responses between solvable and unsolvable tasks. Learning that the caregiver would eventually open the container (by experiencing both attentional states) could have caused the cats to modify their behavior (e.g., seek help earlier or just wait).

A sex-bias was noted in the success rate of passing the acclimation-training-test sequence. Males were more likely than females to make it through the entire study sequence. Takeuchi and Mori (2009) reported in a survey that, regardless of breed, male cats were more apt to seek novelty, while females were more prone to show nervousness during clinic visits. Similarly, in a study validating negative responses of cats to restraint, female cats jumped off the examination table sooner than males after a mock veterinary examination (Moody et al. 2018). Female cats were also shown to be less social towards conspecifics than male cats (Ha and Ha 2017), and more likely to develop separation anxiety in the owner’s absence (Schwartz 2002). Feline dimorphic reproductive strategies may explain the prevalence of boldness-related personality traits in male cats (Dards 1983; Crowell-Davis et al. 2004). However, there is another possible explanation for the observed sex-bias. Treats were used in our study during pre-selection, acclimation, training, and testing. Not all cats respond to treats equally. Vitale Shreve et al. (2017), in a free-operant preference assessment, showed that cats preferred social interaction (50%) over food (37%). The male cats in our study may have had a stronger preference for treats than the female cats and may have been more willing to cooperate with the technician because of that. The use of social reward or toy as a reinforcer could have led to a different male to female ratio of cats being successful in passing the entire protocol sequence.

Age did not have an effect on the successful completion of training and testing; the proportion of younger and older cats passing was 39.3% and 38.5%, respectively. However, younger cats were more attentive to caregivers and less attentive to treats compared to older cats, regardless of the caregiver’s attentional state or test type. In general, younger cats are more active and explorative than older cats (Vogt et al. 2010), and this may explain why younger cats in our study engaged more with their caregivers via showing behaviors.

In summary, in our study cats used different behavioral strategies depending on the situation they faced and the attentional state of the caregiver. During the unsolvable condition, cats increased gaze alternations, spent less time in contact with the caregiver, and they approached the treat container less often. When the caregiver was attentive, cats took their first gaze at her faster, looked at her more often, and approached the treat container more often. We also observed an interaction between the test type and the attentional state of the caregiver: when the caregiver was inattentive, cats showed less sequential behaviors, but only during the unsolvable condition. Our results challenge the popular notion that cats are independent and not people-oriented, and add to the emerging scientific literature that provides evidence that cats can form attachment bond with humans (Vitale et al. 2019), have successful vocal communication with humans (McComb et al. 2009; Nicastro 2004; Saito and Shinozuka 2013; Saito et al. 2019; Takagi et al. 2019), can recognize attentional states in humans ((Mertens and Turner, 1988; Ito et al. 2016; Vitale and Udell 2019), and use human-directed cues (Miklósi et al. 2005; Kraus et al. 2014; Pongrácz et al. 2019). Our results suggest that cats are attuned to their socio-cognitive environment, address intentional behavior at humans to access resources out of their reach, and take into account the attentional availability of humans. In fact, it appears that the attentional state of the owner could serve as important reinforcement for cat-human communication and the establishment of a strong bond between the two.

## Supplementary Information

Below is the link to the electronic supplementary material.

Supplementary file1 (DOCX 25 kb)

Supplementary file2 (XLSX 24 kb)

## Data Availability

Data can be provided as supplementary documents upon request. SAS code for the study can be provided upon request.
