# Supplementary material for: Feline communication strategies when presented with an unsolvable task: the attentional state of the person matters
Source: Anim Cogn. 2021 Apr 2;24(5):1109–19. doi: 10.1007/s10071-021-01503-6 (PMC8360888; doi:10.1007/s10071-021-01503-6)
Supplement: Supplementary file 1 — Supplementary file1 (DOCX 25 kb) [file 10071_2021_1503_MOESM1_ESM.docx]

Table1. Subject information

| Pets | Sex  Spayed (SF)/  Neutered (NM) | Weight (Kg) | Age  (years) | Caregiver | Stage | Attentive (Y) / Inattentive (N) |
| --- | --- | --- | --- | --- | --- | --- |
| 1 | SF | 3.847 | 4.82 | Emily | Fail Acclimation |  |
| 2 | SF | 4.348 | 8.25 | Emily | Fail Acclimation |  |
| 3 | SF | 4.555 | 11.52 | Emily | Pass test | Y |
| 4 | NM | 5.961 | 9.21 | Emily | Pass test | N |
| 5 | NM | 4.277 | 9.72 | Emily | Pass test | Y |
| 6 | NM | 5.761 | 10.25 | Emily | Pass test | N |
| 7 | NM | 4.401 | 8.97 | Emily | Pass test | Y |
| 8 | SF | 5.03 | 7.92 | Emily | Fail Acclimation |  |
| 9 | NM | 5.14 | 5.99 | Emily | Pass test | Y |
| 10 | NM | 4.994 | 11.52 | Emily | Fail Acclimation |  |
| 11 | NM | 5.441 | 3.43 | Emily | Pass test | N |
| 12 | SF | 3.277 | 7.44 | Emily | Fail Acclimation |  |
| 13 | SF | 4.62 | 7.44 | Emily | Fail Acclimation |  |
| 14 | SF | 5.125 | 8.8 | Emily | Fail Acclimation |  |
| 15 | NM | 6.735 | 6.99 | Emily | Pass test | Y |
| 16 | NM | 5.476 | 8.27 | Emily | Pass test | N |
| 17 | SF | 5.814 | 6.28 | Leslie | Pass test | N |
| 18 | NM | 5.527 | 1.99 | Leslie | Pass test | N |
| 19 | NM | 5.869 | 5.61 | Leslie | Pass test | Y |
| 20 | NM | 5.845 | 11.14 | Leslie | Pass test | N |
| 21 | NM | 5.051 | 11.02 | Leslie | Fail test |  |
| 22 | NM | 5.369 | 11.14 | Leslie | Pass test | Y |
| 23 | NM | 6.046 | 10.22 | Leslie | Pass test | Y |
| 24 | NM | 4.871 | 11.14 | Leslie | Pass test | N |
| 25 | NM | 5.315 | 9.98 | Leslie | Pass test | Y |
| 26 | SF | 4.638 | 9.31 | Leslie | Fail Acclimation |  |
| 27 | SF | 4.172 | 11.59 | Leslie | Pass test | N |
| 28 | SF | 3.97 | 5.58 | Leslie | Pass test | Y |
| 29 | NM | 5.292 | 11.58 | Leslie | Pass test | N |
| 30 | NM | 5.879 | 7.44 | Leslie | Fail Acclimation |  |
| 31 | NM | 4.742 | 11.21 | Leslie | Fail training |  |
| 32 | SF | 3.757 | 10.14 | Leslie | Fail Acclimation |  |
| 33 | SF | 4.172 | 6.33 | Emily | Fail Acclimation |  |
| 34 | SF | 4.548 | 5.04 | Emily | Fail Acclimation |  |
| 35 | SF | 4.343 | 11.59 | Emily | Fail test |  |
| 36 | NM | 6.004 | 9.96 | Emily | Pass test | N |
| 37 | NM | 4.933 | 8.97 | Emily | Fail Acclimation |  |
| 38 | NM | 5.627 | 11.42 | Emily | Pass test | Y |
| 39 | SF | 4.531 | 9.33 | Emily | Fail Acclimation |  |
| 40 | SF | 4.892 | 10.86 | Emily | Fail training |  |
| 41 | NM | 5.78 | 7.33 | Leslie | Fail Acclimation |  |
| 42 | NM | 5.656 | 10.58 | Leslie | Fail Acclimation |  |
| 43 | SF | 4.994 | 5.42 | Leslie | Fail Acclimation |  |
| 44 | NM | 5.8 | 8.00 | Leslie | Fail Acclimation |  |
| 45 | NM | 5.82 | 9.33 | Leslie | Pass test | N |
| 46 | NM | 4.984 | 3.00 | Leslie | Fail Acclimation |  |
| 47 | NM | 5.9 | 4.50 | Leslie | Fail Acclimation |  |
| 48 | NM | 6.038 | 5.58 | Leslie | Fail Acclimation |  |
| 49 | NM | 5.626 | 4.58 | Emily | Fail test |  |
| 50 | SF | 4.18 | 3.75 | Emily | Pass test | Y |
| 51 | NM | 5.84 | 3.75 | Emily | Pass test | N |
| 52 | NM | 5.684 | 5.67 | Emily | Fail Acclimation |  |
| 53 | NM | 5.19 | 3 | Emily | Pass test | Y |
| 54 | SF | 4.895 | 7.90 | Emily | Fail Acclimation |  |
| 55 | NM | 5.198 | 1.53 | Emily | Fail Acclimation |  |
| 56 | SF | 4.191 | 5.29 | Emily | Fail Acclimation |  |

Table 2. Main treatment effect of all measures

| Measure | Test Type | | | | | | Attentional state | | | | | | Age | | | | | |
| --- | --- | --- | --- | --- | --- | --- | --- | --- | --- | --- | --- | --- | --- | --- | --- | --- | --- | --- |
|  | Solvable  (LSmeans) | SE | Unsolvable  (LSmeans) | SE | Statistics | P | No Gaze  (LSmeans) | SE | Gaze  (LSmeans) | SE | Statistics | P | Age<=7  (LSmeans) | SE | Age>7  (LSmeans) | SE | Statistics | P |
| Dur_Gaze at Container | 0.87 | 0.24 | 1.17 | 0.24 | -0.92 | 0.37 | 1.27 | 0.26 | 0.77 | 0.25 | 1.38 | 0.18 | 1.04 | 0.29 | 1.00 | 0.22 | 0.12 | 0.90 |
| Dur_Gaze at Caregiver | 4.78 | 0.78 | 5.83 | 0.78 | -1.18 | 0.25 | 3.88 | 0.92 | 6.74 | 0.88 | -2.26 | 0.03 | 5.74 | 1.01 | 4.88 | 0.80 | 0.66 | 0.52 |
| Dur_Interaction with Container | 8.68 | 1.98 | 10.78 | 1.98 | -0.76 | 0.44 | 9.37 | 2.03 | 10.09 | 1.96 | -0.26 | 0.80 | 7.51 | 2.25 | 11.95 | 1.77 | -1.54 | 0.13 |
| Dur_Contact with Caregiver | 20.50 | 4.47 | 14.14 | 4.47 | 2.09 | 0.05 | 18.20 | 6.01 | 16.44 | 5.79 | 0.21 | 0.83 | 26.18 | 6.65 | 8.46 | 5.24 | 2.08 | 0.05 |
| Dur_in Proximity to Container | 22.84 | 3.82 | 24.16 | 3.82 | -0.28 | 0.78 | 22.36 | 4.26 | 24.64 | 4.10 | -0.39 | 0.70 | 19.25 | 4.71 | 27.75 | 3.71 | -1.41 | 0.17 |
| Dur_in Proximity to Caregiver | 34.13 | 5.25 | 32.27 | 5.25 | 0.54 | 0.58 | 36.27 | 7.08 | 30.12 | 6.83 | 0.63 | 0.54 | 42.96 | 7.83 | 23.44 | 6.18 | 1.95 | 0.06 |
| Freq_Gaze at Container | 1.10 | 0.26 | 1.52 | 0.26 | -1.3 | 0.21 | 1.60 | 0.29 | 1.02 | 0.27 | 1.47 | 0.16 | 1.56 | 0.32 | 1.06 | 0.25 | 1.24 | 0.22 |
| Freq_Gaze Alteration | 0.33 | 0.16 | 0.75 | 0.16 | -2.67 | 0.01 | 0.65 | 0.20 | 0.43 | 0.19 | 0.8 | 0.43 | 0.62 | 0.22 | 0.46 | 0.17 | 0.59 | 0.56 |
| Freq_Gaze at Caregiver | 5.37 | 0.60 | 5.49 | 0.60 | -0.16 | 0.87 | 4.74 | 0.68 | 6.11 | 0.66 | -1.45 | 0.16 | 5.86 | 0.76 | 4.99 | 0.60 | 0.9 | 0.38 |
| Freq_Sequential Behvaior | 4.84 | 0.56 | 4.38 | 0.56 | 0.76 | 0.45 | 4.40 | 0.67 | 4.82 | 0.64 | -0.45 | 0.65 | 4.76 | 0.74 | 4.46 | 0.58 | 0.31 | 0.76 |
| Freq_Container to Caregiver | 2.88 | 0.34 | 2.26 | 0.34 | 1.41 | 0.17 | 2.40 | 0.36 | 2.74 | 0.35 | -0.68 | 0.51 | 2.72 | 0.40 | 2.43 | 0.32 | 0.56 | 0.58 |
| Freq_Caregiver to Container | 1.64 | 0.31 | 1.37 | 0.31 | 0.81 | 0.42 | 1.35 | 0.37 | 1.65 | 0.36 | -0.57 | 0.57 | 1.42 | 0.41 | 1.58 | 0.33 | -0.3 | 0.77 |
| Freq_in Proximity to Container | 2.62 | 0.24 | 1.89 | 0.24 | 2.56 | 0.02 | 1.85 | 0.27 | 2.66 | 0.26 | -2.12 | 0.04 | 1.77 | 0.30 | 2.74 | 0.24 | -2.49 | 0.02 |
| Freq_in Proximity to Caregiver | 3.99 | 0.54 | 3.14 | 0.54 | 1.48 | 0.15 | 3.05 | 0.65 | 4.07 | 0.63 | -1.13 | 0.26 | 3.50 | 0.72 | 3.63 | 0.57 | -0.14 | 0.89 |
| Freq_Vocalization | 2.01 | 0.92 | 1.47 | 0.92 | 0.64 | 0.52 | 0.65 | 1.17 | 2.84 | 1.12 | -1.36 | 0.19 | 1.63 | 1.29 | 1.86 | 1.02 | -0.14 | 0.89 |
| Latency_Gaze at owner | 27.03 | 4.69 | 17.28 | 4.57 | 1.51 | 0.14 | 34.26 | 4.62 | 10.05 | 4.72 | 3.68 | 0.00 | 21.78 | 5.21 | 22.54 | 4.18 | -0.11 | 0.91 |
| Latency_ in Proximity to Container | 8.69 | 2.74 | 9.83 | 2.74 | -0.76 | 0.46 | 13.32 | 3.77 | 5.20 | 3.63 | 1.56 | 0.13 | 12.26 | 4.17 | 6.25 | 3.28 | 1.13 | 0.27 |

The unit for duration (Dur) is second and for frequency (Freq) is time.
